# Exercise-induced oxidative stress and melatonin supplementation: current evidence

**DOI:** 10.1186/s12576-021-00812-2

**Published:** 2021-09-01

**Authors:** Joanna Kruk, Basil Hassan Aboul-Enein, Ewa Duchnik

**Affiliations:** 1 Faculty of Physical Culture and Health, University of Szczecin, Szczecin, Poland; 2 Faculty of Public Health and Policy, London School of Hygiene and Tropical Medicine, London, UK; 3 Department of Aesthetic Dermatology, Pomeranian Medical University, Szczecin, Poland

**Keywords:** Melatonin, Bioactivity, Oxidative stress, Inflammation, Exercise, Supplementation

## Abstract

Melatonin possesses the indoleamine structure and exerts antioxidant and anti-inflammatory actions and other physiological properties. Physical exercise can influence secretion of melatonin. Melatonin is used as a natural supplement among athletes to regulate sleep cycles and protect muscles against oxidative damage. Despite decades of research, there is still a lack of a comprehensive and critical review on melatonin supplementation and physical activity relationship. The aim of this literature review is to examine the antioxidant, anti-inflammatory and other biological functions played by melatonin with reference to the effect of physical exercise on melatonin secretion and the effect of this compound supplementation on exercise-induced oxidative stress in athletes. Evidence shows that intense exercises disturb antioxidant status of competitive athletes, whereas supplementation with melatonin strengthens antioxidant status in trained athletes in various sports as the compound showed high potency in reduction of the oxidative stress and inflammation markers generated during intense and prolonged exercise.

## Background

Melatonin (MT) *N*-acetyl-5-methoxytryptamine is an endogenous indoleamine which controls crucial physiological processes such as human circadian rhythms sleep–wake cycle, anxiety, immune, and cardiac function [1–5]. MT influences appetite and regulates insulin levels among other functions [6]. The compound and its metabolites are potent antioxidants which exhibit anti-inflammatory properties and protect mitochondria from damage by scavenging reactive oxygen species (ROS), and reactive nitrogen species (RNS) [7–9]. MT and its derivatives stimulate several antioxidant enzymes activity [1, 10]. Thus, MT plays an important role in maintenance of cellular redox homeostasis and in the aging processes. Increase in free radicals’ level and oxygen non-radical species production, followed by their accumulation in cells, may lead to a disturbance in cellular redox balance; a phenomenon called oxidative stress (OS). This state is characterized by an imbalance between pro-oxidants generation and antioxidant system’s defense against ROS/RNS and efficiency of the DNA repair system [11].

Intense physical activity (PA), similarly as other unhealthy lifestyle factors such as smoking, alcohol, improper diet, or environmental factors (e.g., radiation, viruses, and bacteria), disturbs the redox homeostasis toward oxidation [12, 13]. Intense and prolonged exercise induces inflammation, due to high generation of free radicals and ROS/RNS and possible oxidative muscle damage [14, 15]. In contrast, regular moderate-to-vigorous exercise generates moderate concentration of ROS/RNS, followed by adaptative responses favorable for the organism and exert beneficial effects on onset and progression of a number ROS/RNS-associated diseases [16–18]. The redox homeostasis in the skeletal muscle is key, in the context of sports, because the redox state depends on efficacy of ROS generation [16].

There is growing evidence that physical exercise (PE) may exert both rapid and delayed (12–24 h) effects on human MT secretion [19, 20].

In a previous article, we presented the enhancing effect of PE on catecholamines (CATs) secretion and on cellular redox homeostasis [21]. Evidence has shown that one of the CATs, noradrenaline (NA), is involved in the control of MT synthesis. Due to antioxidant properties MT and its metabolites, and the compound prescription in sleep disturbances of children and adolescents in neuropsychiatric disorders [22] and common ingestion by athletes, presenting the current state of knowledge on association between MT and PE is warranted. The potential of MT as an antioxidant to supplement against negative effects of exhaustive and/or high intense PE or to improve athlete’s performance is necessary. Despite increasing research in this field, there is a lack of a comprehensive critical summary of the research on impact of MT supplementation to prevent against the PE-induced OS. Therefore, this article presents a representative cross-section of evidence in this area. The aim of this paper is to present the effect of PE on MT secretion and to demonstrate the current evidence on the beneficial role of the indolamine supplementation against OS generated in the human body by strenuous exercise. This review also focuses on mechanisms and determinants of exercise-induced OS and summarizes the literature data on biological functions performed by MT, especially with regard to antioxidant and anti-inflammatory activities.

### Chemical structure and biological activities of melatonin and its metabolites

MT, a multifunctional and the most effective antioxidant among natural antioxidants, is endogenously produced in plants and mammals as well as supplied to the body through fruits and vegetables [1]. The compound is synthetized from tryptophan, mainly in the pineal gland in the brain (about 80%) in darkness. Also, other sources of MT in mammals are recognized: bone marrow cells, retina, skin, platelets, lymphocyte, and the gastrointestinal tract [2, 22]. Four enzymatic steps of MT biosynthesis have been detailed: hydroxylation, decarboxylation, acetylation, and methylation [2, 23]. In the first step, tryptophan is metabolized by tryptophan hydroxylase 1 to 5-hydroxytryptophan. Next, the intermediate is converted by the enzyme 5-hydroxytryptophan-decarboxylase to 5-hydroxytryptamine (serotonin). Further, serotonin in mammals is metabolized to *N*-acetyl-5-hydroxytryptamine by serotonin *N*-acetyltransferase and methylated by the enzyme *N*-acetylserotonin *O*-methyltransferase to MT [23].

It is important to note that the intermediate compound in the MT biosynthesis—serotonin is a key neurotransmitter having a wide spectrum of functions in the human body [24]. For example, serotonin plays an essential role in behavior and regulation of central nervous system. MT modulates several functions, such as anxiety, psychological stress, learning, sleep, mood, pain, appetite, and exhibits antioxidant and antidepressant properties, among others. MT is carried by the systemic circulation to central and peripheral tissues (e.g., liver, pancreas, lung, kidney, heart, and the fetal adrenal gland) where functions are organized through a 24-h cycle (so-called circadian rhythms). Moreover, the neurohormone receptors are present in several central and peripheral tissues, e.g., heart and coronary blood vessels, adrenal gland, lung, kidney, prostate, skin, T and B lymphocytes, and adipocytes.

### Chemical structure of melatonin and its metabolites

**Fig. 1:**
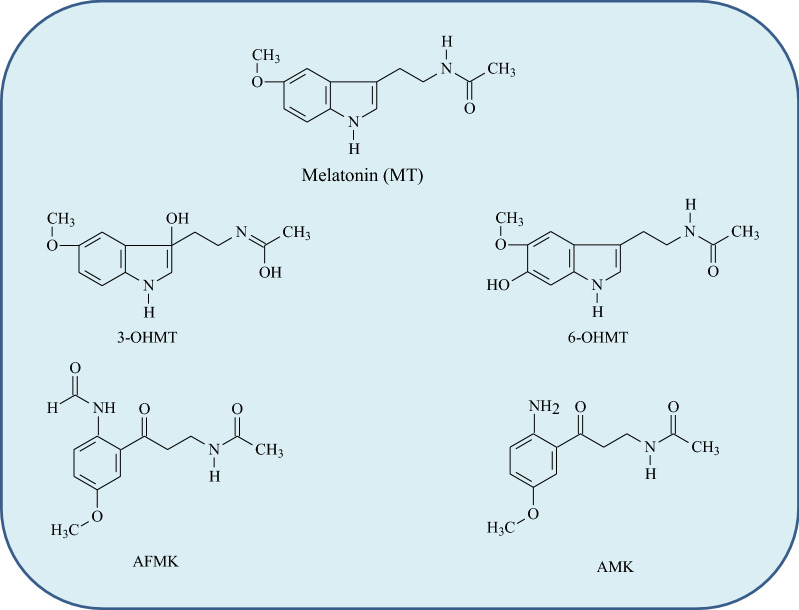
Chemical structures of melatonin (MT) and its main metabolites: 3-OHMT (3-hydroxymelatonin); 6-OHMT (6-hydroxymelatonin); AMK (*N*^1^-acetyl-5-metoxyknuramine); AFMK (*N*^1^-acetyl-*N*^2^-formyl-5-metoksyknuramine)

Melatonin contains an electron-rich aromatic indole heterocycle with methoxy group attached to C5 atom and amide group attached to C3 atom from indole ring [7, 25, 26] (Fig. 1).

The presence of an indole ring allows MT to function as an electron donor, thus, to reduce level of electrophilic species in cells. The site chains enable MT to exhibit both hydrophilic and lipophilic properties and diffuse across the cell membranes [27], thus, to protect biomolecules, such as cytosol, mitochondria, and nucleus against oxidation [1, 9]. In addition, the methoxy and amide groups enhance free radicals’ scavenging ability of the electron-rich indole moiety, and the electronic structure of indole ensures high resonance stability and its reactivity toward ROS [8, 9, 28]. Evidence showed that C-atoms in the indole ring structure are appropriate sites for hydroxyl radical (HO^⋅^) and nitric oxide radical (NO^⋅^) acceptation due to low energetic barrier, followed by adducts formation. Evidence simultaneously has suggested several mechanisms responsible for the MT interaction with free radicals [29] as follows: (a) an electron transfer reaction; this interaction leads to the MT cation formation which exhibits long lifetime and may react with ${\text{O}}_{2}^{ \cdot - }$; (b) hydrogen atom donation, this capacity has been ascribed to the NH group; (c) radical addition reaction, this process is highly efficient with powerful electron acceptors efficacy; in this way MT may deactivate two HO radicals; (d) substitution reaction, this process occurs on carbon atoms, the C-atoms at position 2, 3 or 7 of the indole ring are easily substituted by HO^⋅^; (e) nitrosation reaction, in this process *N*-nitrosomelatonin is generated as a result of the neurohormone reactivity towards NO.

Several distinctive activities of MT have been reported due to its chemical structure. Briefly, evidence showed enhancing DNA repair by affecting genes participated in DNA damage [30, 31]. These properties enable MT to scavenge peroxyl radical (ROO^⋅^), singlet oxygen (^1^O_2_) and peroxynitrite (ONOO‾), and to inhibit the activity of NO synthase and hypochlorous acid (HClO), among other toxic radicals and species [1, 32]. The basic property that distinguishes MT from other commonly applied antioxidants is its ability to scavenge up to ten of ROS/RNS, thus MT is much more potent antioxidant than the classic antioxidants, e.g., GSH or vitamins C and E which scavenger at most one radical [25]. Another distinctive property is transformation of MT on oxidative and enzymatic pathways to several metabolites, which are widely recognized as good free radical scavengers [7, 32]. The main metabolites of MT generated during its reaction with ROS/RNS and/or on the enzymatic pathway, possessing antioxidative properties, include 3-OHMT, 6-OHMT, AMK, and AFMK as are shown in Fig. 1 [25, 28]. The MT metabolites containing hydroxyl group in their chemical structure exhibit lower potency in the scavenging ability than MT and AFMK and AMK metabolites.

### Biological activities of melatonin and its metabolites

**Fig. 2:**
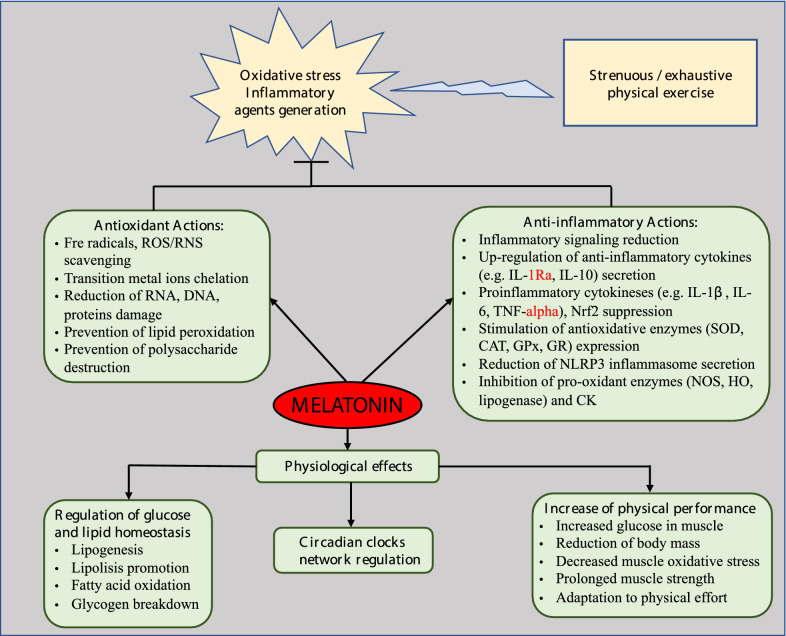
Simplified scheme for the biological functions performed by melatonin

Melatonin and its metabolites exhibit a wide spectrum of both direct and indirect physiological effects in humans [4, 25, 31–34] (Fig. 2). Firstly, these compounds scavenge free radicals and other non-radicals ROS/RNS directly, reducing level of OS, thus show antioxidant abilities preventing inflammation. Secondly, these biomolecules participate in immunomodulation, improve immune defense, and exhibit other physiological activities, e.g., regulate circadian rhythms, body temperature, increase physical performance and glucose uptake in muscles, and prevent against lipid accumulation, among others [22, 35–38].

### Free radical scavenging and anti-inflammatory abilities of melatonin

The main mechanism describing oxygen-based free radical scavenging by MT occurs via electron donation. For example, donation of an electron to HO^⋅^ leads to its deactivation accompanied by the MT transformation to almost non-toxic radical form (indolyl radical cation). Further reactions of indolyl radical cation with HO^⋅^ leads to the formation of several hydroxylated products (e.g., 3-OHMT, 6-OHMT) also having antioxidant properties [7, 25, 26, 29]. Moreover, evidence shows that the AFMK and AMK metabolites (Fig. 1) react with HO^⋅^ in hydrophilic and lipophilic media at diffusion controlled-rates ranging from 1.2 to 7.5 × 10^10^ M^−1^ s^−1^ and with trichloromethylperoxyl radical (^⋅^OOCCl_3_), although they do not show reactivity towards hydroperoxy radical (HOO^⋅^) [32]. The reactivity of MT and its derivatives exhibit continuous protection of biomolecules against OS, reducing concentration of the most dangerous to cell radicals [26, 32]. The order of the relative scavenging activity on the whole is as follows: AFMK > MT > AMK [1], although it is dependent on the environment polarity [26]. In turn, the hydroxylated metabolites of MT, such as 2-HOMT formed during MT reaction with HClO as well as 4-OHMT and 6-OHMT not only directly scavenge free radicals, but also through removing of transition metal ions in the redox state (e.g., Fe^2+^, Cu^+^) diminish concentration of HO^⋅^. Another important antioxidant property of MT is decomposition of H_2_O_2_ and the ability to quench ^1^O_2_ [1, 39].

MT and its metabolites (3-OHMT, AFMK, and AMK) also protect against metal toxicity acting as antioxidant chelating agents on two manners: via direct chelation mechanism and deprotonation reaction linked with metal ion chelation [39]. It was found that the above molecules completely inhibited OS induced by the Cu^2+^—ascorbate mixture, and metabolite 3-OHMT exhibited the highest efficiency [1]. The chelation activity of MT originates from the chemical structure, i.e., presence of two oxygen and two nitrogen atoms acting as di-, tri-, and tetra-dentate ligands with transition metals [40]. MT and its metabolites prevent against a free radical chain reaction of lipid peroxidation where MT and its metabolites act as inhibitors of free radicals initiating the lipid peroxidation, thus protect cells from the harmful effect of the peroxidation products containing a carbonyl group [41].

Further, MT and its metabolites possess ability to reduce chronic and acute inflammation [34]. Although inflammation is a natural response of the human immune system to initiate stress stimuli acute and/or chronic inflammation potentially leads to tissue damage of several organs and possibly to disease [42]. An inflammatory process results from the loss of balance between proinflammatory cytokines and anti-inflammatory agents’ levels towards activation of nuclear factor κB (NF-κB) signaling [43]. The nuclear factor penetrates the nucleus and binds to specific components modulating transcription of proinflammatory genes [42].

MT and its metabolites can exert their anti-inflammatory effect through inhibition of the transcription factors involved in proinflammatory cytokines generation, e.g., activator protein 1 (AP-1), hypoxia-inducible factor-1α (HIF-1α), nuclear factor erythroid 2-related factor2 (Nrf2), NF-κB, prostaglandin synthesis, reduction of production of adhesion molecules, infiltration of neutrophils in tissue, decrease of COX-2 and inducible nitric oxide synthase (iNOS) expression as well as by increasing superoxide dismutase (SOD), CAT, glutathione peroxidase (GPx), and T-helper immune response. These actions of MT reduce inflammation and decrease further ROS formation [22, 40]. For this reason, MT is the antioxidant of great interest for medicinal purposes as OS and chronic inflammation are associated with an increased risk of several cancers [44].

Another noteworthy beneficial property of MT is its capacity to protect against cytotoxicity of heavy metals, such as lead, arsenic, chromium, cadmium, and aluminum, induced through activation of basic regulators of antioxidant inflammatory pathways: NF-κB and Nrf2. Evidence demonstrates their opposing actions: Nrf2 regulates antioxidant pathway via promotion of the transcription of antioxidant enzymes and cytoprotective genes, while NF-κB is the promotor of inflammatory pathways; both transcription factors are regulated by OS [45]. MT induces dissociation of Nrf2 from Keap-1 (Kelch-like ECH-associated protein), which can translocate into the nucleus, where it upregulates and promotes the transcription of antioxidant genes. In addition, Nrf2 can also bind to NF-κB target genes and suppress their transcription [40, 45]. The antioxidative, anti-inflammatory abilities of MT and its metabolites as well its high lipophilicity also predispose this compound to reduce the aging process, and age-related diseases, e.g., neurological Alzheimer’s disease, Parkinson’s disease, or Huntington’s disease, among others [9, 46].

### Anti-tumor effect of melatonin

There is growing scientific evidence that both OS and psychological stress have been linked with alterations in the immune system responses. The disturbance manifests by a decrease in immune natural killer (NK) cells activity and their ability to respond to the proinflammatory cytokines. NK cells and tumor antigen cytotoxic T lymphocytes (CTLs) are important immune anticancer agents of which activity is enhanced by MT. Activation of CTLs by heat shock protein (HSP70), ATP and high mobility group box1 (HMGB1) leads to the secretion of anticancer cytokines, such as interferon gamma (IFN-γ) and TNF-alpha which can induce apoptosis in cancer cells [47]. The hormone also reduces an activity of T regulatory cells (Treg) and cancer-associated fibroblasts of the agents recognized as promoters of the growth and invasion of tumor cells [47]. Cancer cells show over-proliferate ability through modulation of protein expression and the signal transduction pathways such as HIF-1, NF-κBs, phosphoinositide 3-kinase/protein kinase B (P13K/AKT), insulin-like growth factor receptor (IGF-1R), cyclin-dependent kinases and estrogen receptor signaling [48]. MT possess the ability to selectively block the signaling pathways of tumor cells participating in metastasis by modulation of cell–cell and cell–matrix interactions and can restrain the proliferation of cancer cells and inhibit their growth [48, 49]. An important property of MT is an ability of regulation of P13K/AKT intracellular signaling pathway which is involved in the promotion of metabolism, growth, proliferation, cells survival, and angiogenesis. The P13K/AKT pathway is overactive in many cancers reducing cells apoptosis [48]. Although the precise molecular mechanisms that would allow MT to affect tumor initiation and progression are not fully understood, several other beneficial effects have been demonstrated, e.g., participation in maintaining the genomic integrity, due to its antioxidant and inflammation activities, inhibition of androgen and estrogen formation and activity and also their receptors, dysregulation of tumor metabolism [47, 48, 50]. The latter activity of MT results from its capacity to switch glucose metabolism in cancer cells from the glycolytic anaerobic category in the cytosol to a normal oxidative phosphorylation for ATP synthesis in the mitochondria and coverts pyruvate to acetyl-coenzyme A. In this way, MT can decrease energy supply needed for ATP formation and limit a rapid cancer cells growth and proliferation [44, 51, 52]. Evidence has demonstrated that in tumor cells glycolytic anaerobic metabolism definitely overweight the oxidative metabolism; pyruvate formed in cytosol during glucose metabolism is not transformed to the mitochondria but is metabolized to lactate in cytosol [Warburg effect]. The Warburg effect limits the efficient production of ATP and citrate, which could arrest glycolysis as in the normal cells, and, in addition, it prefers intracellular alkaline environment, enhances glycolysis and cells cycle progression, cancer cell growth, aggressiveness, resistance to apoptosis, and makes cancer therapy difficult [51, 52]. Evidence showed that the MT effect is concentration dependent as cancer cells showed the highest cytostatic glycolysis during the day and nighttime exposure to light [51]. Findings have documented the oncostatic role of MT against several types of cancer (breast, prostate, oral, gastric, lung, brain, ovarian, colorectal, liver, renal) [50, 53]. The compound has also been shown to be useful in cancer treatment during chemotherapy and radiotherapy [48, 54, 55]. The benefit in the MT application in cancer therapy results from its different effects on tumor cells and the normal cells. The use of MT in chemotherapy allows to reduce the dose of applied drugs to destroy tumor cells through their increased sensitivity on drugs interaction which limits their resistance [56]. In addition, MT decreases side effects of the cancer chemotherapy, increases rate of patients survival and improves overall well-being. Similarly, combination of MT with radiotherapy has also been found to restrict damage of the normal tissue related to radiation dose and achieve greater cancer inactivation; MT plays role of a radiosensitizer [54, 55].

### Other physiological function of melatonin

Evidence documents that the primary physiological function of MT is to delivery information about the diurnal cycle of light/darkness to the human body and synchronization of central and peripheral oscillators distributed in tissues and organs [5, 57]. The circadian clock system plays a key role in maintenance of homeostasis and human health. The circadian time keeping system is composed of a generator of circadian rhythms located in suprachiasmatic nuclei (SCN) of the hypothalamus which synchronizes the peripheral cellular clocks with daily periodic environmental changes [19]. This MT action as a time indicator to the biological clocks allows humans rapidly adept themselves to environmental conditions and survive. This function is important as the circadian rhythm disturbance can increase the risk of metabolic, cardiovascular, and mental diseases, as well as cause poor quality of life [1, 2]. The circadian rhythm of the hormone synthesis and secretion is closely related to the rhythm of sleep [58]. MT stabilizes circadian rhythms and exerts the chronobiotic effects acting on the plasma membrane trough G protein-dependent receptors type 1 and type 2 called MT1 and MT2, and its rhythmic release is regulated by a central circadian rhythm generator [57, 59]. Density of MT1 and MT2 receptors in the SCN is high, although the receptors are also distributed in peripheral tissues and organs (e.g., adipose tissue, liver, heart). The signals generated by SCN induce the MT synthesis in darkness, and the process is controlled by SCN via the sympathetic nervous system (SNS) [57].

Importantly, MT is effective in adjusting sleep–wake cycles and improving the quality of sleep and is commonly used as a medicine for the treatment of sleep problems, e.g., in insomnia, jet leg [60]. With age, the production of MT decreases [22] which may have an influence on poor sleep quality in elderly individuals and cause the prevalence of comorbid the mental and physical health disorders. The severity of sleep impairments correlate with the decrease of endogenous MT formation and its concentration in cerebrospinal fluid [22]. It is important to note that several MT receptor agonists can also mimic MT activation, thus they are able to regulate the body sleep–wake rhythm through their actions on the MT receptors MT1 and MT2 in the SCN. Ramelteon, agomelatine and tasimelteon are representative MT receptor agonists used for sleep disorders [58, 60]. It is maintained that the MT receptor agonist connecting to the MT1 receptor subtype can directly improve sleep latency and quality, whereas the agonist action on the MT2 receptor subtype positively influences the circadian system, e.g., by promoting early sleep. Also, the use of the MT receptors to treat comorbid insomnia in several health disorders (neurological, metabolic, cardiovascular, psychiatric) is of great importance [60–62]. The MT receptors agonists, similarly as MT, have an influence on several signaling pathways, exhibit free radical scavenging ability, stimulate the activity and/or expression of antioxidant enzymes, and suppress the proinflammatory cytokines release. For example, Wang et al. [61] reported that ramelteon administration in C57BL/6 mice cells elevated traumatic brain injury, prevented against OS and inflammation. In this study, treating with the MT agonist significantly increased the expression of IL-4, IL-10, SOD, GSH, GSH peroxidase and decreased levels of IL-1β, TNF-α and MDA. The authors also observed that ramelteon decreased KEAP1 expression of a key sensor of oxidative and electrophilic stresses, promoted Nrf2 nuclear accumulation, and increased levels of heme oxygenase-1, that is Nrf2 regulated gene having antioxidant and anti-inflammatory effect in vascular cells. Ramelteon also controlled the redox controlling enzyme NQO1 (NAD(P)H: quinone oxidoreductase 1) activity. NQO1 plays significant role in quinone metabolism and exhibit the defense against OS [63]. Further, the reduced expression of proinflammatory cytokines (IL-6, IL-1β, TNF-α), glial fibrillary acidic protein and protection against increased expressions of proinflammatory COX-2 and against a potent inflammatory mediator prostaglandin E_2_(PGE_2_) as well as excessive NO^⋅^ production were reported [62].

In addition to well-established functions outlined above, MT has also been identified as an important promotor of a healthy metabolic rate through glucose homeostasis re-establishment and lipid metabolism regulation [36]. These physiologic effects of the indolamine have drawn great attention in the recent years [4, 5]. Evidence shows MT can regulate glycogen breakdown to glucose, preserve glycogen stores, improve insulin resistance in the skeletal muscle and liver, and reduce obesity, protecting from several diseases caused by glycogen storage [6]. These MT properties influence physical performance during PE of high intensity [64]. Although evidence on the beneficial metabolic effects of MT is reported and findings showed that the hormone exhibits very low side effect [4], there is no full agreement in this respect (as reviewed by Garulet et al. [37]). Detailed discussion of the physiologic activities may be found in the reviews [4, 5, 36].

## Physical exercise and melatonin

Proper sleep and recovery are essential to athletes’ benefit as well as enhancing their physical performance from training programs. Evidence has accumulated showing that athletes commonly use sleep-enhancing supplements, and MT is one of the most used aid in this respect, due also to other positive effects on the body, e.g., as an antioxidant in protection of muscles against OS and enhancer of performance [19].

### Influence of physical exercise on cellular redox state

The effect of PE on the cellular redox homeostasis has been widely studied since the 1970s, especially regarding exercise of moderate intensity (50–< 60% $V{\text{O}}_{2}^{\max }$) and exercise of vigorous/high intensity (≥ 60% $V{\text{O}}_{2}^{\max }$). Independent of exercise type and intensity, exercise ≥ 50% $V{\text{O}}_{2}^{\max }$ induces an increase of ROS/RNS concentration above physiological level, and their concentration is dependent on several factors (e.g., exercise determinants, postural position during exercise, state of training, age, gender, and diet) [65]. During exercise non-mitochondrial sources of ROS are the main contributor to overall amounts of generated species in the skeletal muscle, while during rest and recovery the mitochondrial source is decisive [66].

There is significant evidence-based research carried out during the past four decades that regular exercise of moderate intensity is a key factor regulating the level of ROS/RNS in a cell protection against chronic cell exposure to these species if their level is low [67, 68]. ROS/RNS are formed constantly in various tissues even at rest as a part of normal metabolic processes. They play role of secondary messengers in intracellular signaling, participate in gene expression, support cellular proliferation and relaxation of vascular smooth muscle cells, and regulate angiogenesis, among others [12, 69]. Primarily, PE increases mitochondrial metabolism and ROS/RNS production, followed by an increase in stress resistance and is responsible for variety of physiological adaptations (e.g., mitohormesis) [70]. Further, regular moderate-to-vigorous PE regulates the energy expenditure, insulin-like growth factor-1 (IGF-1), and insulin-like growth factor binding proteins (IGFBPs) balance, defenses against inflammation, and enhances the immune system functioning. Exercise improves metabolism of all the human biological systems and cells as well as tissues tolerance to higher concentration of ROS generated during exercise training [71], enhancing activity of antioxidant enzymes in muscles and mitochondrial biogenesis [9, 10].

Besides the beneficial effect of PE on humans, evidence shows acute bouts of long-lasting and high-intensity endurance exercises can cause biochemical and hormonal disturbance, generate ROS/RNS at the rate exceeding the endogenous antioxidant system performance and disrupting redox homeostasis, i.e., induce OS in untrained subjects [72]. In addition, diet poor in antioxidants enhances the stress. Under such type of exercises the maximal consumption of molecular oxygen in the muscle fibers may reach 100-fold increase compared with the rest state. Then, skeletal muscles produce large amounts of free radicals, ROS and RNS, including HO^⋅^ and NO^⋅^, and are considered as the major source of these toxic species [14, 65, 66]. The increased production of the reactive species in muscles includes mainly mitochondrial nicotinamide adenine dinucleotide phosphate (NADPH) oxidase, phospholipase A2, and reactions catalyzed by xanthine oxidase (XO), such as a breakdown of ATP accompanied by generation of adenosine diphosphate (ADP) and adenosine monophosphate (AMP) and further hypoxanthine via the adenylate kinase reaction [13, 14, 73]. Evidence summarizes possible adverse effects of acute exercise and/or overtraining, such as DNA/RNA and protein damage, increased lipid peroxidation, decreased natural killer (NK) cells function and T cell activities, increased angiogenesis, induction of protooncogenes, activation of transcription factors, alternation in the muscle genome, the immune system damage, and change of signal transduction [14, 66]. Acute reduction of the muscle action and their fatigue was described to high level of ROS produced during muscle contraction [15, 65].

The damaging effect of OS has been confirmed experimentally by identifying changed level of OS markers in blood and during muscle biopsies [74–76]. These processes may lead to chronic inflammation and increase a risk of several civilization diseases, including cancer development, progression and metastasis (more detailed reviews on this topic are available, e.g., Ref. [71]). Evidence for the adverse oxidative modifications of muscular biomolecules were mainly reported after acute or chronic anaerobic exercise [74, 77].

Acute high-intensity PE can cause biochemical and hormonal disturbance, increase OS, being positively correlated with overexpression of proinflammatory cytokines, such as interleukin-1 (IL-1), interleukin-6 (IL-6), tumor necrosis factor-alpha (TNF-α) and CRP [15]. Further, large changes in immune system activity and lipid and carbohydrate metabolism following exhaustive exercise can promote acute muscle inflammation and tissue damage [71]. Additionally, endurance athletes often experience poorer quality of sleep, due to high training load [78]. Intense training also decreased athletes endogenous MT level [79]. On the other hand, recent studies demonstrated that the redox signaling pathway participates in the chronic response of the skeletal muscle to endurance training [15, 79, 80]. This muscle activity includes an uptake of glucose, influences on insulin sensitivity, mitochondrial biogenesis, and initiation of antioxidant enzymes [81, 82].

### Effect of physical exercise on melatonin and serotonin secretion

#### Melatonin secretion

Synthesis and secretion of MT is stimulated in darkness and occur with circadian rhythm. Evidence has indicated that the hormone concentration increases between 9:00 and 10:00 p.m. reaching a maximum value between 3.00 and 4.00 a.m. following by a decrease between 7:00 and 9:00 a.m. On average, the MT levels are estimated to be about 5 pg/mL during the day and 50–100 pg/mL at the night [83]. Synthesis and release of MT are modified by several factors (genetic factors, sex hormones, age, diet, light exposure, season, hypoxia, PE, diseases, drugs) [83–85]. These factors may potentially act as enhancers of MT secretion or its inhibitors. Briefly, increases in the MT production and its secretion were reported for a diet containing MT or the compounds supporting its synthesis, e.g., tryptophan, vitamins, and minerals that act as activators or co-factors during the hormone synthesis [85]. Further, extension of the dark phase during winter, regularly taking antidepressants and MAO inhibitors also increase the MT secretion [84]. Conversely, the presence of light, especially the nocturnal exposure to artificial light of blue cyanin color, use of β1-adrenergic blockers and non-steroidal anti-inflammatory drugs, chronic alcohol consumption, and hypoxia have inhibitory effects. Additionally, MT release is decreased during aging and in patients with long-term chronic diseases, such as dementia, type 2 diabetes mellitus, rheumatoid arthritis, cancer [86]. Accumulated evidence presents the effect of PE on human MT secretion as exercise induces an upregulation of many cell processes and physiological functions, including the circadian clocks network synchronization [19, 20, 87]. Studies that tested the magnitude and direction of the circadian rhythm phase shift induced by PE showed that PE can both reduce and increase the MT concentrations, according to the rhythm phase shifting (advanced or delayed) or remain without effect. This interaction depends on the time of exercising during the day, the intensity of light, training level, and exercise determinants (intensity, duration, dose), regardless exercise type (aerobic, anaerobic, concentric, resistive) and gender [88]. Two modes of the exercise effect on human MT release have been observed rapid and delayed, depending on its duration and daily repeatability [20].

Exercising in the late evening during ascending phase of circadian MT release was accompanied by delaying the hormone secretion, or even inhibition, depending on exercise duration and intensity. In turn, when MT levels reached the maximal physiological value, even exercise of high intensity performed in early evening might enhance MT release. There is a suggestion that exercising near the offset of MT release or during the daytime (morning, afternoon) did not exert significant changes in plasma MT concentration. Further, among the circadian clock system external stressors, which can interact or cooperate with PE and modify neural activity and/or the circadian genes expression and change the clock time are: light, psychological stress, food, and PE. For this reason, it is difficult to control all factors affecting the MT release, and the data from literature are not yet consolidated. The major stimuli responsible for the stressors influence on the circadian clocks include glucocorticoids, sympathetic nerves, OS, pH changes, decreased oxygen levels in the local tissues, changed cytokines level, and temperature [89]. Evidence has shown ROS activate the redox signal transduction in the SCN and exhibit day–night variation controlling neuronal membrane excitability. Physical exercise alters sympathetic neural activity and increases MT release [19] caused by increased noradrenaline secretion during the sympathetic system stimulation [21].

Table 1 presents the representative data for the MT secretion following PE. The studies varied in many respects, as exercise duration (short time-from 1 to 8 days, long-lasting-from 2 to 12 months), exercise type, exercise intensity (moderate, moderate-to-vigorous, acute until exhaustion), training conditions (hypoxic, normoxic conditions, indoor training, outdoor training), sex, and measuring the timing of maximal secretion of MT as well as the measurement methods. An increase of MT concentration following PE in the morning [90] and in hypoxic conditions [91], decreases in the same [79, 92], and a lack of the effect [93, 94] were reported. Referring to the MT measurement timing, Carlson et al. [90] observed that exercise performed in the morning resulted in elevated MT secretion by 20% compared to the hormone secretion induced by the afternoon exercise. The representative studies listed in Table 1 have some limitations, which have influenced their results. These limitations include the lack of control for the major factors influencing MT secretion, the small number of measurement time points for MT detection or the small sample size.

**Table 1:** The effect of physical exercise on melatonin levels in humans

Study, year	Participant characteristics	Category, timing of PE and MT detection	Main results	Author’s conclusion
Thrift et al. 2014 [93]	RCT (51 men, 49 women and 51 men and 51 women as a stretching control (average age 40–75 years, previously sedentary)	12-month program of moderate-to-vigorous exercise (60 min daily, 6 days/week at 60–80% $V{\text{O}}_{2}^{\max }$) MT detection: baseline and 12-month follow-up urinary metabolite of MT, 6-sulphatoxymelatonin	No statistically significant changed concentrations of 6-sulphatoxymelatonin after 12-months exercises vs controls (p = 0.66). Baseline metabolite levels were significantly higher in women compared to men, but not after exercise	Moderate-to-vigorous 12-month exercise did not change level of 6-sulphatoxymelatonin
Kilic et al. 2016 [94]	Ten healthy, sedentary males (average age 22.2 ± 0.24 years)	Strenuous exercise as acute exercise until exhaustion, according to the Bruce protocol (Cosmed T150 treadmill test). Blood MT detection at rest, at 10:00 a.m. and immediately after exercise as well as after 48 h at 12.00 p.m. (rest) and immediately after exercise	No statistically significant change in serum MT levels after exercise performed during day or night vs the levels at rest: (3.63 ± 0.08 vs 3.37 ± 0.18 and 4.41 ± 0.26 vs 4.33 ± 0.21, pg/mL, respectively)	Exhaustion PE did not affect the level of MT in the blood, independently on exercise timing (daytime, at night)
Zarei et al. 2016 [92]	Thirteen healthy, non-athletic males (average age 19–23 years)	20 min daily of moderate-intensity exercise (running, 50–60% $V{\text{O}}_{2}^{\max }$). Two months exercise from 9:30 to 9:55 p.m., two months silent MT measurements: 24 h prior to exercise, 48 h after exercise, two months after the last exercise	Significantly decreased MT levels in PBMCs with exercise: post-exercise 7.94 ± 0.35 pg/mL, 2-month silent 6.05 ± 0.27 pg/mL vs pre-exercise (9.16 ± 0.19 pg/mL). Significantly increased IL-17 secretion by 39% in the post-exercise time	Long-lasting engaging in moderate-intensity exercise caused decrease in MT release, and increase in IL-17 cytokine level
De Aquino Lemos et al. 2018 [91]	RCT (*n* = 40) healthy men randomized into four groups: normoxia (*n* = 10), hypoxia (*n* = 10), exercising under normoxia (*n* = 10), exercising under hypoxia (*n* = 10), (average age 22 ± 3 years). Observation period 36 h	Aerobic moderate exercise on a treadmill at 50% of VT1 for 60 min, performed under normoxia and hypoxia conditions from 11:00 a.m. to 12:00 noon blood MT detection: at 7:30 a.m. (the 1st and 2nd days), at 10:30 p.m. (the 1st and 2nd nights)	Significantly increased nocturnal blood MT levels in the hypoxia group vs the normoxia group after the second night; both values were lower than those in the exercise group under hypoxia	PE under hypoxia enhances nocturnal level of MT, influences its daytime level, and improves sleep quality
Carlson et al. 2019 [90]	12 healthy males, regularly exercising, runnerslage (average age 20.7 ± 0.62 years)	Three protocols: 30 min of steady state running on a level treadmill at 75% $V{\text{O}}_{2}^{\max }$ morning exercise (9.00 a.m.), afternoon exercise (4:00 a.m.), no exercise. Salivary MT detection: 8:00 p.m., 10:00 p.m., 3:00 a.m. following exercise	Significantly increased levels of MT at 03:00 a.m. compared with those at 8:00 and 10:00 p.m. after completed all the protocol session. MT level at 10.00 p.m. was significantly, elevated (by 20%) after morning exercise vs afternoon exercise	Exercising in the morning may increase MT release compared with exercise performed at afternoon
O’Donnell et al. 2019 [79]	Ten elite female netball athletes (average age 23 ± 6 years)	Athletes one netball training session over a 7-day period and one rest day (control). Mean heart rate during training—145 ± 10 bpm, mean rating of perceived exertion—14 ± 1 according to the Borg scale MT detection: immediately pre-training, 7:15 p.m. and post-training 10:00 p.m. and during control day	Significantly decreased salivary MT levels in pre-training (6.2 pg/mL) and post-training (17.6 pg/mL) vs a rest day (14.8 and 24.3 pg/mL), respectively)	Training caused significantly decreased levels of MT

*MT* melatonin, *PE* physical exercise, *PBMCs* peripheral blood monocular cells, *RCT* randomized controlled trial

These findings remain in accordance with previous findings because there is no clear consensus in the literature on the effect of PE on endogenous profile of MT secretion [19].

It is noteworthy to present the quantitative impact of some factors influencing the PE–MT release association. For example, earlier experimental studies of Buxton et al. reported both increases [20] and decreases [95] of MT levels following exercise depending on time of day and on intensity, duration, and type of exercise. The authors found that during exercise in nighttime, when the MT level is elevated and intensity of exercise is high, the hormone increase may reach 50%. Moreover, the MT secretion was found to be dependent on individual’s postural changes. These researchers maintained that moderate and high intensity exercise may influence MT levels and involve the circadian clock phase-shifting effects. Also, a study by Carr et al. [96] confirmed that plasma level of MT was transiently increased in women during all acute submaximal exercise tests of progressive endurance training after 30-min exercise completion. A previous study by Theron et al. [97] showed that additional factors, such as BMI, lighting conditions, the time of day of exercising, and age may influence the relationship between plasma MT concentration and PE. The authors demonstrated increased plasma MT level in adult Black males who in the step-climbing exercise generated an energy output of 185 W/m^2^ body surface area, immediately after PE and 1 h after. Evidence indicated a threefold increased MT mean level in group exercising under 320 lx lighting (*n* = 15) and a 2.84-fold under 54 lx lighting (*n* = 15). Evidence exists that the season of year (winter, summer) also affects the PE–MT association, and changes of MT concentration after exercise vs before exercise may depend on the initial level of individual’s endogenous MT concentration [19]. An interesting observation done by Serrano and co-workers showed professional road cyclists experienced of an adaptation to physical overloads during sports competitions [98]. It is maintained that this ability allows them to regulate intracellular OS, limiting an induction of the proinflammatory cytokines. Moreover, elevated levels of MT detected after competition, resulting from increased activity of SNS can give evidence that the protective effect of exercise operates via modulatory effect on MT secretion [57, 99]. Although processes responsible for the PE effect on MT secretion are not fully understood, several mechanisms are proposed. As maintained above, one of the biogenic catecholamines—noradrenaline participates in regulation of MT biosynthesis by the mammalian pineal gland [100]. Evidence presented high increases of noradrenaline level (2 × 10^3^%) in professional cyclists and even sevenfold increase after half-marathon runs [101, 102]. Exercise stimulates SNS activity and catecholamines (dopamine, noradrenaline) release [21], thus it can modulate MT secretion, followed by a net phase-shifting through the pineal gland and SCN of the hypothalamus, which causes an expression of receptors for MT. Further, PE stimulates the midbrain raphe nuclei activating serotoninergic input to the intergeniculate leaflets, which take part in the circadian function regulation through SCN [57]. It is suggested that an initiation of activity of the serotoninergic neurons from the median raphe nuclei is excited by serotonin and depends on its concentration in SCN [103], thus serotonin, a compound linked with well-being regulates the mammalian circadian rhythmicity.

### Serotonin response to exercise

The body of evidence from animal models and human studies demonstrated the beneficial effect of exercise on brain functions [104, 105]. Regulations of the neurotransmitters is one of several exercise’s effect on brain function, among others [104]. Evidence exist that PE modulates serotonin system and can promote increased concentrations of serotonin, as muscle activation needs higher amounts of tryptophan [106]. The authors have suggested that exercise reduces levels of amino acids competing with tryptophan through muscle intake and make these amino acids easier to cross the blood–brain barrier into muscles. In this way, serotonin and MT synthesis and their levels are increased in the brain dependently on exercise intensity. The ability of PE to modulate the activity of catecholaminergic and serotonergic systems and to enhance the brain function show that exercise as a positive activating agent of body’ stress response. Additionally, aerobic exercise has a high ability to decrease depression and anxiety levels and to improve physical performance [105].

Several recent reviews have summarized experimental findings on the serotonin response to PE [104, 107, 108]. Most serotonin changes following exercise have been well documented in rodents, but only a limited number of human studies have been conducted. A review of 22 studies in rats by Meeusen and De Meirleir presented that the majority of analyzed studies found changes in synthesis and metabolism of serotonin or its metabolites and CATs in response to exercise [107]. Another review by Basso and Suzuki [108] has analyzed research findings on the effect of exercise intensity and dose on serum serotonin in humans and rodents. The authors have suggested that acute long-term exercise increased serotonin levels and its metabolites in the frontal cortex, hippocampus, striatum, and midbrain as well as dopamine in rodents. These reviews have maintained that the serotonin increase in the central nervous system of rodents requires appropriate intensity of exercise, and the amount of serotonin released is positively correlated with PE intensity. Further, the findings of studies conducted in humans show that individuals engaged in acute exercise of low intensity may benefit from significant decreases in depression and anxiety disorders in contrast to high-intensity acute exercise, which is considered to contribute to fatigue. In this regard, it is maintained that a value of exercise-induced ratio of serotonin to dopamine and noradrenaline plays a significant role in the development of fatigue and performance [107, 108]. The authors suggested that interaction between brain serotonin and dopamine during acute PE can be responsible for the brain region specific changes in levels of serotonin.

Statistically significant increases in blood serotonin levels dependent on PE have also been reported in a few experimental studies in humans and rodents in the last decade [109–111]. A study by Valim et al. [109] showed that women with fibromyalgia (*n* = 22) randomly divided into two groups aerobic walking exercise and stretching exercise three times/week for 20 weeks experienced statistically significant increase of serum serotonin concentration in response to aerobic exercise. Stretching exercise resulted in statistically insignificant increase of serotonin level. Also, a study by Arazi et al. [110] noticed aerobic exercise significantly increased levels of serotonin and dopamine in the physically active group of 16 men addicted to opium in comparison to those who do not exercise (*n* = 18). The active group was engaged in aerobic exercise—walking 2–3 times/week for 20–30 min. The results show that even low-intensity aerobic exercise can affect the levels of these neurotransmitters. Recently these findings have been confirmed by Matsunaga et al. in rats (*n* = 48) [111]. The researchers studied the effect of intensity and timing of forced exercise (running on a motorized wheel), voluntary exercise (free running on a wheel) and low-dose exercise (voluntary, running exercise limited to 1 h), through 4 weeks on levels of serotonin. They found that response of serotonin to exercise was exercise dose-dependent; significantly increased levels of serotonin and its metabolite (5-hydroxyindoleacetic acid) were seen in the dorsal and median raphe nuclei in rats engaged in forced exercise. Also, increased concentrations of the neurotransmitter were found in the paraventricular hypothalamic nucleus and caudate putamen in voluntary exercised rats. Low-dose exercise was without effect on serotonin levels, and the heavy exercised rats experienced increased anxiety-like behavior.

## Role of melatonin supplementation in response to exercise-induced oxidative stress

Exogenous compounds exhibiting antiradical and antioxidant potency play a key role in regulation of ROS/RNS concentrations. Under OS conditions, endogenous concentration of MT are unable to prevent damages to biomolecules by ROS. Evidence shows supplementation with appropriate antioxidants may improve the cellular redox homeostasis, decrease oxidative modification of DNA bases, lipids, and proteins, and decrease muscular fatigue of athletes, thus enhance exercise performance [112, 113]. It is known that antioxidant supplementation is common practice among endurance athletes who hope to minimize OS and enhance physical performance [49, 66, 114], and MT is a universal antioxidant that enjoys great popularity among athletes.

Table 2 lists the representative studies (*n* = 11) for the effect of MT ingestion on exercise-generated OS and inflammation.

**Table 2:** Characteristics of the representative epidemiological studies on the effect of melatonin supplementation on exercise-induced oxidative stress in humans

Study, year	Participant characteristics	MT supplementation	Exercise measured	Results	Conclusions
Ochoa et al. 2011 [115]	Highly trained performing regular exercise amateur athletes (*n* = 20): experimental group (*n* = 10) supplemented with MT and control group (*n* = 10) receiving placebo	Oral intake of five tablets of 3 mg MT: one tablet two days before the exercise test, three tablets on the previous days and one tablet 1 h before beginning the test	High intensity constant run with several degrees of high effort on total distance 50 km with permanent climbing, altitude changing from 640 to 3393 m	Significantly increased blood levels of TNF-α, IL-6, IL-1Ra, urine 8-OHdG and isoprostane concentrations in both tested groups. Efficiently reduced lipid peroxides, TNF-α and 8-OHdG before and after exercise in the MT group vs placebo group	MT supplementation can reduce muscle damage via modulation of OS and preventing overexpression of proinflammatory cytokines
Maldonado et al. 2012 [116]	16 young male football players (experimental group *n* = 8, control group *n* = 8)	Experimental group treated with 6 mg MT, control group treated with placebo, 30 min prior to exercise	Acute sport training of high intensity (heart rate 135 beats per minute)	Exercise increased MDA in both groups, but significantly decreased in MT group after 60 min of training. Decreased triglyceride and increased serum IgA levels after training in MT group	Supplementation with MT in acute sport training decreased the OS generated by exercise, enhanced the serum TAS, and improved metabolism of lipids
Leonardo-Mendonça et al. 2017 [122]	Randomized double-blind study of 24 resistance trained students (males). MT-treated men *n* = 12, placebo-treated men *n* = 12 (control group)	Experimental group supplemented with MT (100 mg daily, 30 min before bedtime for 4 weeks)	8 sessions a week (about 10 h/week): 5 sessions-resistance training (3 sessions between 60–75% of maximal strength and 2 sessions between 80 and 90% of maximal strength), 2 sessions—weight training and 1 session—aerobic running	Increased ORAC levels by MT vs placebo after exercise. Reduced LPO, iNOS, GSSG/GSH and GPx/GRd ratios, CK, LDH, creatinine, cholesterol. Prevention against AOPP increase in MT group vs placebo group	MT enhanced potency of the endogenous antioxidant system, restored redox equilibrium state and protected against OS damage
Ortiz-Franco et al. 2017 [117]	14 male healthy athletes (age: 20–37 years) engaged in a 2-week randomized, double-blinded trial (MT-treated group and placebo-treated group)	20 mg MT/day or placebo administered before exercise during the controlled study period (MT group)	Training program combined strength and high intensity interval trainings (6 sessions/week 60–75 min/day. $V{\text{O}}_{2}^{\max }$: 70%, 90%)	Significantly increased MT level, TAC and GPx levels, decreased DNA damage in MT-treated group vs placebo group after 2-week exercise	MT treatment strengthens antioxidant state of athletes and protects DNA from damage caused by high intensity exercise
Ziaadini et al. 2017 [118]	Two groups of sedentary women: involved in exercise training and treated with MT (*n* = 10) and only training (*n* = 10), mean age: 24.2 ± 1.03 and 23.4 ± 1.83 years, respectively	3 mg/day MT supplementation before exercise training	8-week (3 days/week) exercise training of increasing intensity and volume from 60 to 80% HRmax through 15 to 45 min	Significantly increased levels of MDA after long-lasting aerobic exercise training. Suppression of post-exercise increased MDA in the exercising and supplemented group	Supplementation with MT may decrease ROS levels, thus improve lipid profile
Beck et al. 2018 [119]	11 males moderately active, mean age: 24.18 ± 3.92 years	MT (6 mg) or placebo ingestion 30 min before exercise	Exercise on cycloergometer with initial workloads of 75 W and increments of 15 W each 3 min till exhaustion Maximal aerobic capacity 120.88 ± 18.78 W	A time to exhaustion significantly lower in placebo group compared to that with MT administered by approximately 19%	MT supplementation enhanced aerobic tolerance but was without effect on the biochemical and hematological parameters
Brandenberger et al. 2018 [120]	Ten cyclists long-distance training, mean age 25.0 ± 4.0 years	5 mg MT administered 15 min before time trial. Controls: placebo 15 min before time trial	32.2 km cycling time trial performance at $V{\text{O}}_{2}^{\max }$ 62.7 ± 6.8 (mL kg^−1^ min^−1^) on ergometer. Mean powers (190.4 ± 40.4 W and 190.0 ± 45.7 W, respectively)	No statistically significant differences between both groups in duration (completion times: MT group 64.94 ± 5.95 min, placebo group 65.26 ± 6.85)	Supplementation of MT did not exhibit of significant effect on performance in thermoneutral environment
Czuczejko et al. 2019 [123]	Professional athletes: 47 football players, 19 rowers, 15 adults non-training males (control group)	5 mg MT administered before sleep through 30 days in the preparatory period for athlete’s competition	Athletes: exercise on a cycle ergometer at 75% $V{\text{O}}_{2}^{\max }$	Decreased blood MT levels in footballers and rowers vs controls before MT intake. Increased serum MT level in footballers and in rowers after a 30-day MT intake. Reduced OS markers: MDA, IL-6, CRP, and low-density lipoproteins	Supplementation of MT in professional athletes during intense training may protect against the toxic action of ROS/RNS and inflammation
Souissi et al. 2018 [121]	Eight healthy moderately trained male students, mean age: 21.8 ± 0.9 years	6 mg MT supplementation or placebo at 09:00 a.m. in a randomized order 50 min before exercise	Running at 60% $V{\text{O}}_{2}^{\max }$ for 45 min on a treadmill, starting at a speed of 8 km/h and increasing by 0.5 km/h after every minute	Exercise elevated inflammatory markers: CRP, LDH, ALAT, ASAT in both placebo or MT intake groups	MT ingestion before moderate prolonged submaximal exercise showed no anti-inflammatory action
Cheikh et al. 2020 [124]	Randomized double-blind trial of 14 healthy-trained male athletes, mean age 154 ± 0.3 years	10 mg MT or placebo ingestion (control) after vigorous late-evening exercise (10:00 p.m.)	Two-test sessions (separated at least one week) Running-Based Anaerobic Sprint Test at 8:00 p.m. and in the following morning (7:30 a.m.)	Reductions of: WBC, NE, LY, CRP, muscle and hepatic damage enzymes (CK, ASAD), LDH, MDA and homocysteine before and after strenuous exercise vs placebo group	MT intake after strenuous late-evening exercise diminished transient leukocytosis and protected against lipid peroxidation and muscle damage in teenage athletes
Farjallach et al. 2019 [125]	20 soccer players mean age 18.81 ± 1.3 years, MT group (*n* = 10), placebo group (*n* = 10)	Nocturnal oral MT (5 mg) or placebo ingestion in a double-blind manner	Intensive 6-day training-repeated sprint ability test: sprints 6 × 40 m with a 20 s of passive recovery between repetitions	Decreased resting OS markers: AOPP, leukocytosis and CK. Decreased post-exercise leukocytosis and markers of cellular damage (CK, ASAT, ALAT), increased GPx and GR activities in MT-treated group vs placebo group	Nocturnal MT intake during intensive training decreased OS, leukocytosis, cellular damage, and improved exercise performance

*LY* lymphocytes, *WBC* white blood cells, *NE* neutrophils, *CRP* C-reactive protein, *CK* creatine kinase, *LDH* lactate dehydrogenase, *ASAT* aspartate aminotransferase, *MDA* malonaldehyde, *LDL* low-density lipoprotein, $V{\text{O}}_{2}^{\max }$ maximal oxygen uptake, *ALAT* alanine aminotransferase, *CHO* formaldehyde, *ORAC* oxygen radical absorption capacity, *LPO* lipid peroxidation, *GSH* glutathione, *GSSG* glutathione disulphide, *AOPP* advanced oxidation proteins products, *MT* melatonin, *GPx* glutathione peroxidase, *GR* glutathione reductase, *TAC* antioxidant status, *iNOS* inducible nitric oxide synthase, *IL-1Ra*, interleukin-1 receptor antagonist, *8-OHdG* 8-hydroxy-2′-deoxyguanosine

These observational studies differ from one another mainly in terms of MT timing: taking MT before exercise [115–121] or before bedtime [122–125] and its dose, exercise type, intensity, duration and timing, and training level. The most common types of sport training in these studies are cycle ergometer, treadmill exercise or running, in which individuals mainly engaged in maximal or submaximal exercise. The authors performed measurements of OS the markers (leukocytosis, MDA, 8-OHdG, TNF-α, IL-6, LDL, AOPP), inflammatory markers (CRP, ALAT, ASAD), TAS as well as of other endogenous antioxidants, using mainly the blood sapless. The data indicate MT intake reduced the markers of OS and inflammation in athletes engaging in severe high-intensity exercise, independently of timing of MT intake. Only three [119–121] of 11 presented experimental studies examined the MT influence on redox status followed moderate-intensity exercise. These authors found no effect of the hormone (5–6 mg doses) on biochemical and hematological parameters. This finding is in line with the evidence that at least strenuous exercise and/or overload training cause excessive production of ROS/RNS and an increase of OS [64].

As presented above, in addition to antiradical, antioxidant activities and enhancing antioxidant enzymes potency, the hormone can change glucose metabolism from glycolytic anaerobic category to a normal aerobic mitochondrial oxidative phosphorylation for ATP synthesis. An ingestion of MT before exercise increases use of glucose as a substrate energy. During prolonged exercise, glucose and fatty acids are utilized followed by a reduction of glycogen in muscle and liver. Evidence has shown that MT intake before PE preserves muscle glycogen stores of the compound which limits exercise performance [19].

Three of 11 presented studies [119, 120, 125] also examined effects of MT supplementation on exercise performance, finding its improvement in the two reported studies [119, 125]. In turn, a study by Souissi et al. [121] tested the effect of MT supplementation on inflammation induced by exercise at 60% $V{\text{O}}_{2}^{\max }$, i.e., the lowest value of the maximum rate of O_2_ consumption for exercise classified as high intensity. The authors found increased levels of inflammatory markers in both the MT-supplemented group and the placebo group and no statistically significant differences between these groups.

A few studies presented in Table 2 examined also endogenous MT level in athletics after high-intensity training, observing significantly increased concentrations of inflammatory markers (TNF-α, IL-6, IL-1Ra), isoprostane and 8-OHdG [115], malonaldehyde (MDA) [116, 118] or decreased blood levels of MT [116, 123], compared with control groups. Explaining the inconsistency of the results may originate from different training status of sportsmen, training protocols, and variability of their redox state dependently on endogenous antioxidants secretion.

It is worth noting that some studies on the effect of strenuous exercise on cellular homeostasis observed changes only in some OS markers, and even their lack [15, 126]. Consistent with this finding, the current review by Powers et al. [10] has presented a shape of a curve illustrating the association between muscle fiber levels of ROS production induced by PE and physiological function of these species. The curve exhibits biphasic bell shape (hormesis, parabolic shape), with maximum of physiological benefit corresponding to limited muscle fatigue and exercise of moderate-to-vigorous intensity. This part of hormesis describes the normal physiological range of ROS, generated in the skeletal muscles by exercise and is considered as an important action to adapt of individuals to endurance training. In turn, the second phase of an association, described by the descending part of hormesis curve, starts from the curve maximum, and ends in the point corresponding zero of physiological functions, despite a further increase in exercise-induced ROS generation. This part of hormesis suggests that further increase in ROS level caused by prolonged high-intensity exercise does not result in tissue damage, assuming that it is probably that PE exerts a true hormetic effect on the body [10].

In addition to MT supplementation, the level of endogenous MT was seen to be dependent on daytime performing PE and its dose. Although supplementation with MT showed high potency in reduction of OS markers formed during intense exercise, the evidence is insufficient to recommend specific dose of this antioxidant. These observations are in accordance with findings of other authors discussed earlier and below. Briefly, there is clear consensus in the literature that noradrenaline is involved in biosynthesis of MT and can determine its concentration [100]. Increased secretion of the catecholamine in plasma was observed above lactate threshold (on average of 50–80% of athletes’ $V{\text{O}}_{2}^{\max }$) [21, 68] and was dependent not only on the level of training and duration, but also on an individual’s psychological stress, health state and muscular mass engaged in training [68]. An experimental study by Kim and Kim [127], included 8-week-old rats that were subjected to high-intensity (*n* = 30) and low-intensity (*n* = 30) exercises for 15 min daily over 4 weeks, demonstrated greater increase in blood MT concentration in rats exercising with high-intensity exercise, compared to MT levels before and after high- and low-intensity exercise. The authors have maintained that MT secretion can be raised by exercise of high intensity, while exercise of low intensity can exert pro-oxidative effect resulting in decreased MT level due to the hormone interaction with stressors. This may explain, at least partially, the differences in findings reported in Table 2. In turn, a review by López-Flores et al., [128] of the research published in 2000–2018 (18 articles) on the effect of MT intake on sport performance underlined an important role not only a MT dose, but also daytime the supplement intake corresponding to the circadian clock system. The researchers found that ingestion of MT up to 1 h before PE did not improve athletic performance. The authors maintain that a dose of 10 mg of MT consumed 30 min before sleep may increase the sport performance briefly. It was also suggested that supplementation with higher concentrations of this antioxidant at the time of sport training may show adverse effects, e.g., sleep deprivation and the SNS suppression. There is increasing scientific evidence, mainly based on use of vitamins C and E, lipoic acid, and coenzyme Q, showing that antioxidant supplementation at high doses may be unfavorable for sport training adaptation because exogenous antioxidants can impair ROS/RNS level in skeletal muscle during sport training [129]. A previous experimental study in vitro indeed showed that MT at high concentration may also exert prooxidant activity [130]. The authors observed an increase of the amount of HO^⋅^, ${\text{O}}_{2}^{ \cdot - }$ and ^1^O_2_ generated in the Fenton-like reaction in the presence of high concentrations of MT. Further, animal’s study by Hong and colleagues reported that MT treatment with nanomolar doses of rats with the advanced knee osteoarthritis for 4 weeks alone or combined with moderate treadmill exercise (30 min/day) prevented periarticular muscle damage and cartilage degeneration [131]. The authors maintain that these beneficial effects occurred through the circadian clock system, due to restoring of clock-controlled genes and correction of the abnormal chondrocyte phenotype. They observed higher reduction of serum TNF-α when MT intake was combined with exercise. However, prolonged MT administration to rats resulted in promotion of the proteolytic cleavage of receptor activator of nuclear factor kappa-B ligand (RANKL) protein in the synovium, followed by severe subchondral bone erosion. In turn, an animal study by Gedikli and co-workers [132] included MT injection (10 mg/kg) to Sprague-Dawley rats and studied exposition to intense exercise. The authors found cellular degenerations of kidney and liver tissues in rats engaged in exercise and a decrease in these damages in MT-treated rats. Evidence from rodents’ studies documents the important regulatory and modulatory effects, supporting action of MT supplementation on animals’ tissues, and skeletal muscle exposed to OS stimuli, but these findings have not yet been explored in humans. These findings show that high-dose antioxidant supplements including MT may be linked to health risk.

Another important and often discussed problem in this research area concerns adaptation to OS in sport training. There is no clear consensus in the literature that intake of antioxidants enhances adaptation to resistance exercise training. To explain the research findings incompatibility, Merry and Ristow [129] have suggested the possibility of a dose–response association between ROS/RNS levels and exercise-training adaptation and physical performance as a hormetic response, i.e., a low dose (physiological amounts) of OS stimuli such as ROS/RNS exerts beneficial adaptive response of cells, whereas large doses of the stressors exposition, by contrast, exhibit an opposite effect (inhibition), i.e., a decrease of exercise performance. Both PE and MT can exert the beneficial effect through modulation and activation of stress resistance pathways, among others [133]. The dose–response dependence finds confirmation that both MT and physical endurance training induce antioxidant enzymes activities through increased mitochondrial content concentrations, and the Keap-1–Nrf2–ARE pathway and NF-κB signal transduction as a response to OS and electrophilic stress [134, 135].

## Conclusions

This overview clearly summarizes the dispersed literature findings on MT biosynthesis, its chemical and biological properties, discusses exercise-related redox signaling and intense exercise-induced disturbances in redox homeostasis, provides the current knowledge on the effect of exercise on MT release and the effect of MT supplementation on endurance exercise-induced OS in athletes. The available literature findings confirm an important role of MT and its metabolites as a free radical and ROS/RNS scavengers, protectors of DNA damage, and reducers of the cellular and tissue oxidative damage. Current evidence highlights a wide spectrum of MT anti-inflammatory and antitumor actions, OS reduction, and physiological effects on humans, such as circadian clock network regulation, glucose and lipid homeostasis regulation, and effect on physical performance. Also, utility of MT and its agonists for insomnia treatment has been demonstrated. Strong evidence suggests mitochondria are the central organelles for antioxidant actions of MT. Physical exercise alters SNS activity and MT secretion. The current evidence for the exercise dose–MT response relationship showed conflicting findings, similar to previous findings, indicating the effect of PE on the exogenous MT level in cells is extremely complex due to several factors increasing or decreasing its secretion as well as on dependencies on many factors describing exercise, an individual’s redox state, training status, environmental conditions, MT intake timing, body mass, among others. PE increases concentration of ROS/RNS followed by activation of protein kinases, transcription factors and gene transcription, exerting beneficial adaptation or negative effects on the human body, depending on dose and exposition time of cells to this exercise. Strong experimental evidence is available for the positive effect of MT supplementation on lowering the proinflammatory cytokines, lipid peroxides, C-reactive protein, and ROS/RNS of which level could be strongly elevated in athletes engaged in prolonged endurance exercise, independently on MT supplementation timing. This compound has a potency to increase level of antioxidant enzymes and the GSH/GSSG ratio, thus to maintain the cellular redox homeostasis in athletes engaged in acute, high-intensity sports training. Following this, epidemiological studies have found utility of MT intake to be daytime-, dose-, and exercise-dependent. The associations between PE, OS, and MT supplementation are very complex; physiological level of ROS/RNS and PE of moderate intensity are necessary to stimulate improvement of endogenous antioxidant system action.

Despite a large knowledge base on the beneficial properties of MT, the exact mechanism underlying its effect on exercise-induced disturbance in redox homeostasis are not yet fully understood. Also, the current research is not sufficient to indicate recommendations concerning effective but safe dose of MT intake by sportsmen. Clinical studies are needed to establish optimal MT dose, time of day and duration its supplementation to avoid adversely affecting levels of ROS/RNS signaling and consequently of the negative effect on exercise-training organism adaptation.

As the reported studies, overall, included large variations of exercise program protocols and a small sample of athletes, analyzed different markers of OS, were based on a limited extent factors increasing/decreasing the hormone release, were different in terms of MT and exercise timing and applied different MT doses, future research is needed. New research should include larger samples of athletes and address safe dose of MT intake and safe exercise training programs for different sport categories. In this sense, we hope that findings demonstrated in this review will encourage the continuation of research in this important health topic. The summarized and synthesized data of existing knowledge may help coaching staff to incorporate safe sport-specific training program, MT dose and timing. They may also be useful to research clinicians regarding the use of anti-inflammatory properties of this compound in the treatment of inflammatory diseases, including COVID-19.

## Data Availability

Not applicable.

## Abbreviations

^⋅^OOCCl_3_: Trichloromethylperoxyl radical
^1^O_2_: Singlet oxygen
3-OHMT: 3-Hydroxymelatonin
6-OHMT: 6-Hydroxymelatonin
ADP: Adenosine diphosphate
AFMK: *N*^1^-Acetyl-*N*^2^-formyl-5-metoksyknuramine
AKT: Protein kinase B
ALAT: Alanine aminotransferase
AMK: *N*^1^-Acetyl-5-metoxyknuramine
AMP: Adenosine-5′-monophosphate
AOPP: Advanced oxidation proteins products
AP1: Activator protein 1
ARE: Antioxidant responsive element
ASAT: Aspartate aminotransferase
ATP: Adenosine-5′-triphosphate
CATs: Catecholamines
CHO: Formaldehyde
CK: Creatine kinase
CRP: C-reactive protein
GPx: Glutathione peroxidase
GR: Glutathione reductase
GSH: Glutathione
GSSG: Glutathione disulphide
HIF-1α: Hypoxia-inducible factor-1α
HO^⋅^: Hydroxyl radical
HOO^⋅^: Hydroperoxy radical
IGF-1: Insulin-like growth factor-1
IGFBPs: Insulin-like growth factor binding proteins
IL-1: Interleukin-1
IL-1Ra: Interleukin-1, receptor antagonist
IL-6: Interleukin-6
iNOS: Inducible nitric oxide synthase
Keap-1: Kelch-like ECH-associated protein
LDH: Lactate dehydrogenase
LDL: Low density lipoprotein
LPO: Lipid peroxidation
LY: Lymphocytes
WBC: White blood cells
MDA: Malonaldehyde
MT: Melatonin
NA: Noradrenaline
NADPH: Nicotinamide adenine dinucleotide phosphate
NE: Neutrophils
NF-κB: Nuclear factor κB
NK: Natural killer
NO^⋅^: Nitric oxide radical
Nrf2: Nuclear factor erythroid 2-related factor2
ONOO‾: Peroxynitrite
ORAC: Oxygen radical absorption capacity
OS: Oxidative stress
P13: Phosphoinasitide-3-kinase
PA: Physical activity
PBMCs: Peripheral blood monocular cells
PE: Physical exercise
RANKL: Receptor activator of nuclear factor kappa-B ligand
RNS: Reactive nitrogen species
ROO^⋅^: Peroxyl radical
ROS: Reactive oxygen species
SCN: Suprachiasmatic nucleus
SNS: Sympathetic nervous system
SOD: Superoxide dismutase
TAC: Antioxidant status
TNF-α: Tumor necrosis factor-alpha
*V*O_2_max: Maximal oxygen uptake
XO: Xanthine oxidase
